# Biallelic Loss of Function Variants in Myocardial Zonula Adherens Protein Gene (MYZAP) Cause a Severe Recessive Form of Dilated Cardiomyopathy

**DOI:** 10.1161/CIRCHEARTFAILURE.123.011226

**Published:** 2024-03-04

**Authors:** Juan Pablo Ochoa, Laura Lalaguna, Jesús G. Mirelis, Fernando Dominguez, Esther Gonzalez-Lopez, Clara Salas, Gaston Roustan, Kathryn A. McGurk, Sean L. Zheng, Paul J.R. Barton, James S. Ware, María Victoria Gómez-Gaviro, Enrique Lara-Pezzi, Pablo Garcia-Pavia

**Affiliations:** Hospital Universitario Puerta de Hierro Majadahonda, IDIPHISA, Madrid, Spain (J.P.O., J.G.M., F.D., E.G.-L., C.S., G.R., P.G.-P.).; CIBERCV, Instituto de Salud Carlos III, Madrid, Spain (J.P.O., F.D., E.G.-L., C.S., E.L.-P., P.G.-P.).; Centro Nacional de Investigaciones Cardiovasculares, Madrid, Spain (J.P.O., L.L., F.D., E.G.-L., E.L.-P., P.G.-P.).; National Heart and Lung Institute (K.A.M, S.L.Z., P.J.R.B., J.S.W.), Imperial College London, United Kingdom.; MRC London Institute of Medical Sciences (K.A.M., S.L.Z., P.J.R.B., J.S.W.), Imperial College London, United Kingdom.; Royal Brompton and Harefield Hospitals, Guy’s and St Thomas’ Hospital NHS Trust, London, UK (S.L.Z., P.J.R.B., J.S.W.).; Instituto de Investigación Sanitaria Gregorio Marañón, Madrid, Spain (M.V.G.-G.).; Departamento de Bioingeniería e Ingeniería Aeroespacial, Universidad Carlos III de Madrid, Leganés, Spain (M.V.G.-G.).; Centro de Investigación Biomédica en Red de Salud Mental, Madrid, Spain (M.V.G.-G.).; Universidad Francisco de Vitoria, Pozuelo de Alarcón, Spain (P.G.-P.).

**Keywords:** cardiomyopathies, dilated cardiomyopathy, human genetics, tachycardia, ventricular, whole exome sequencing

Dilated cardiomyopathy (DCM) is the most frequent cause of heart failure in the young and the leading cause of transplantation. Almost half of the cases have a familial (hereditary) component, but even in familial cases, the diagnostic yield of genetic testing is lower than <40%.^1^ *Myocardial zonula adherens protein* (*MYZAP*) gene encodes a protein widely expressed in cardiac tissue, being an emerging candidate to become a DCM-associated gene.

We evaluated a family from Spanish origin (European ancestry) in which 2 sisters were affected by a severe form of DCM with healthy unaffected parents. The study was approved by the Hospital Universitario Puerta de Hierro ethics committee and patients provided informed consent. Data are available from the corresponding author upon reasonable request.

The proband was a 35-year-old female with dyspnea (New York Heart Association II) and palpitations after her first pregnancy. Echocardiography showed mild dilatation of the left ventricle (LV) with systolic dysfunction (LV ejection fraction, 44%) and frequent ventricular ectopic beats (1000/24 h) were detected on Holter ECG. Cardiac magnetic resonance showed biventricular midventricular fibrosis predominantly located in the LV. Familial work-up was performed. Both the 62-year-old father and 60-year-old mother had normal cardiac studies. However, her asymptomatic 32-year-old sister showed LV dilatation with LV ejection fraction of 35% and a similar pattern of late gadolinium enhancement on cardiac magnetic resonance (Figure [A]). Nonsustained VT episodes were detected, and an implantable cardioverter defibrillator was implanted. During follow-up she received an appropriate shock while sleeping due to sustained VT. None of the affected patients had extracardiac features. Initial genetic analysis of desmosomal genes and a next generation sequencing cardiomyopathy panel (126 genes) were negative.

**Figure.**
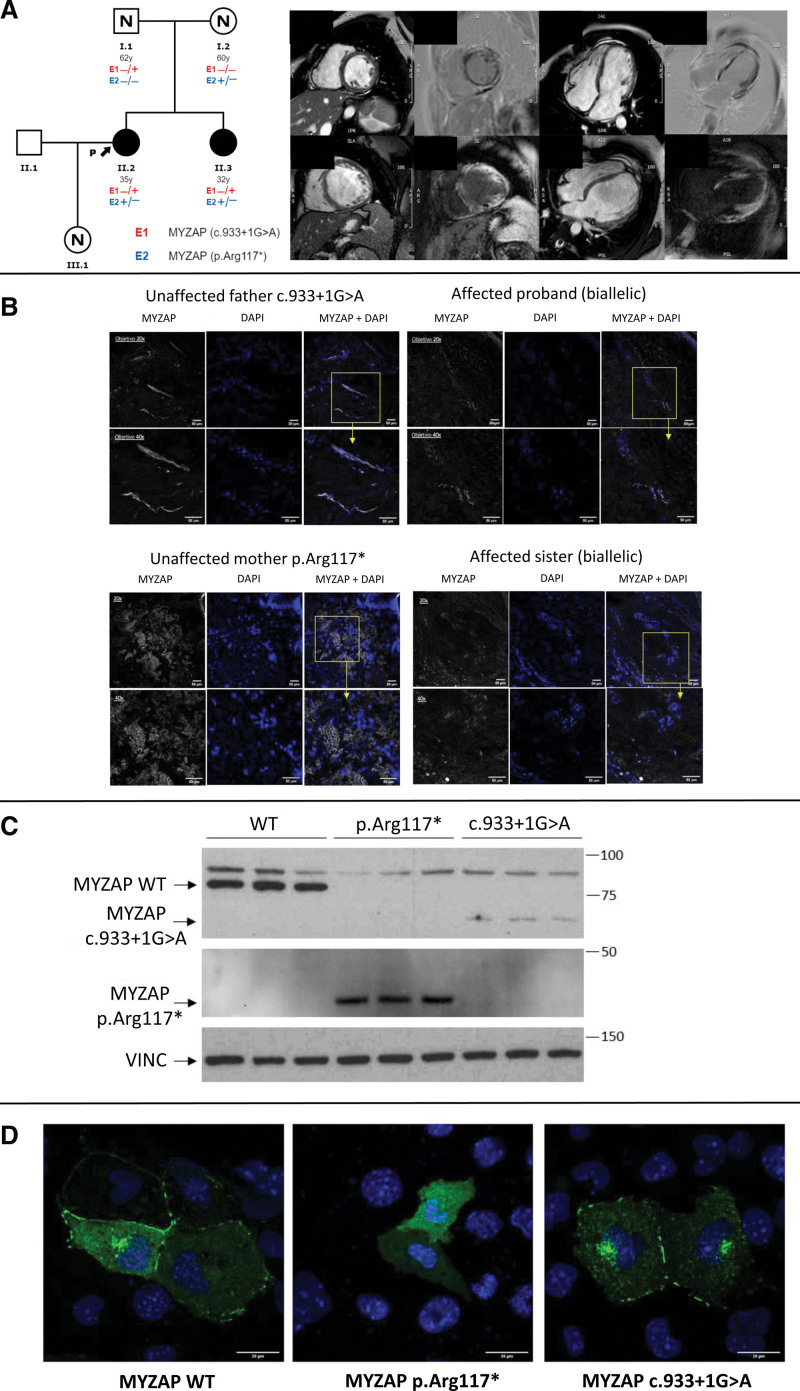
**Clinical and functional evaluations performed in the family. A**, Family pedigree and cardiac magnetic resonance imaging of the index case (**upper**) showing LV dilatation (LVEDV=168 mL) and extensive areas of midventricular fibrosis predominantly in the left ventricle. Her affected sister (**lower**) had similar findings on magnetic resonance imaging (LVEDV=260 mL). **B**, MYZAP immunostaining in skin samples from affected siblings and unaffected parents. Skin biopsies were immunostained with anti-MYZAP antibody (white). Nuclei were stained with DAPI (blue). Images were acquired with a 20× objective. Magnifications of the selected areas (yellow squares) were acquired with a 40× objective. **C**, Western blot analysis of MYZAP in HL-1 cells transfected with MYZAP-WT, MYZAP-p.Arg117*, and MYZAP-c.933+1G>A. MYZAP-c.933+1G>A shows significantly lower expression level of MYZAP compared with MYZAP-WT and MYZAP-p.Arg117*. **D**, Immunofluorescence analysis of localization of MYZAP in transfected HL-1 cells showing that MYZAP-WT and MYZAP-c.933+1G>A are observed predominantly as perinuclear aggregations and in the cytoplasmatic membrane, while MYZAP-p.Arg117* delocalizes being distributed in the cytoplasm but not in any particular structure at cell level. Bar, 20 µm. DAPI indicates 4′,6-diamidino-2-phenylindole; LVEDV, left ventricle end diastolic volume; VINC, vinculin; and WT, wild-type.

Exome sequencing was performed in all family members. Assuming a recessive model, we identified *MYZAP* as the only gene with 2 rare candidate loss-of-function (LoF) variants in both siblings. The first was a nonsense variant (NM_001018100.5 c.349C>T) predicting a premature termination codon (p.Arg117*) in exon 4. The second, NM_001018100.5 c.933+1G>A, affected the first nucleotide of the splice donor site of intron 8, predicting the loss of the donor site. Each parent carried 1 variant (Figure [A]), confirming that they were present in compound heterozygosis (different alleles) in the affected daughters.

We hypothesized that only biallelic LoF variants in *MYZAP* would be associated with DCM. We performed immunostaining of skin samples with anti-MYZAP antibodies in the family to evaluate if there was protein expression as it had been described in pemphigus patients.^2^ A similar distribution of the MYZAP staining pattern was observed in all individuals, which was preferentially located in perivascular areas, but with a weaker expression in samples from carriers of biallelic variants (Figure B).

Cardiac muscle cell lines (HL-1) were transfected with GFP (green fluorescent protein)-tagged expression vectors for MYZAP: peGFP-C1-MYZAP-WT, peGFP-C1-MYZAP-p.Arg117*, and peGFP-C1-MYZAP-c.933+1G>A. Western blot analysis showed a significantly lower expression of MYZAP c.933+1G>A compared with MYZAP-WT and MYZAP-p.Arg117* (Figure [C]). Immunofluorescence to evaluate the subcellular protein localization showed that MYZAP-WT and MYZAP-c.933+1G were predominantly localized in the plasma membrane and as perinuclear aggregates, whereas MYZAP-p.Arg117* was distributed randomly throughout the cytoplasm, without reaching intercellular junctions (Figure [D]). These experiments confirm that neither of the 2 alleles are viable: one of them produces a protein that is degraded and the other produces a dysfunctional protein that is delocalized in the cytoplasm. These results, in which no functional protein was produced in affected carriers, support previous findings of functional studies in zebrafish^3^ and knock-out mice^4^ that develop DCM with decreased contractile function, and increased mortality, being MYZAP localized in the intercalated discs.

Finally, to evaluate the relevance of *MYZAP* variants in humans, we obtained data from 200 571 participants from the UK Biobank and found 29 distinct rare LoF variants in 98 individuals. There were no biallelic LoF carriers and none of the LoF heterozygous individuals were diagnosed with cardiomyopathy. Furthermore, none of 1446 patients with cardiomyopathies studied by whole genome sequencing in the 100 000 Genomes Project England had biallelic LoF variants in *MYZAP*. Cardiomyopathy was not more prevalent in carriers of heterozygous *MYZAP* LoF variants (2/26; 7.7%) compared with individuals without *MYZAP* variants (1420/15 552; 8.4%; χ^2^ test: odds ratio, 0.91 [95% CI, 0.15–3.14]).

In this research, we describe and demonstrate the association between biallelic LoF variants in *MYZAP* and a severe form of DCM, characterized by profuse biventricular fibrosis and ventricular arrhythmias. This phenotype is coincident with 2 homozygous LoF cases recently reported.^5^ Unlike the previous report, affected individuals in our family exhibited LoF in compound heterozygosity, providing further support to *MYZAP* as a recessive DCM-associated gene and suggesting that the relevance of *MYZAP* variants in DCM may be higher than previously anticipated. This could be particularly important in populations with high rates of consanguinity. Furthermore, by analyzing large population cohorts we show that although LoF variants in single heterozygosity in *MYZAP* are observed at low frequency in the general population, they are not associated with cardiac disease in the heterozygous state. Based on our findings, we propose *MYZAP* as a gene to be considered for evaluation in patients with DCM, particularly among gene-elusive patients with arrhythmogenic DCM whose parents do not show cardiac disease.

## ARTICLE INFORMATION

### Acknowledgments

For the purpose of open access, the author has applied a Creative Commons Attribution (CC BY) license to any Author Accepted Manuscript version arising.

### Sources of Funding

This study has been funded by grants from the Spanish Society of Cardiology (basic research grant 2021) to Dr Ochoa, Spanish Ministry of Science PLEC2022-009235 MCIN/AEI/10.13039/501100011033 co-funded by the European Union NextGenerationEU/PRTR to Drs Lara-Pezzi, Gómez-Gaviro, and Garcia-Pavia, and PID2021-124629OB-I00 and TED2021-129774B-C22 to Dr Lara-Pezzi, and from Instituto de Salud Carlos III (ISCIII) DTS22/00030 co-funded by the European Union to Dr Gómez-Gaviro. Drs Lara-Pezzi, Ware, and Garcia-Pavia are funded by the Pathfinder Cardiogenomics Programme of the European Innovation Council of the European Union (DCM-NEXT project; project 101115416). The CNIC is supported by the ISCIII, the Ministerio de Ciencia e Innovación (MCIN) and the Pro CNIC Foundation and is a Severo Ochoa Center of Excellence (grant CEX2020-001041-S funded by MICIN/AEI/10.13039/501100011033). Drs Ware, McGurk, and Barton have been funded by the Medical Research Council (United Kingdom), Sir Jules Thorn Charitable Trust (21JTA), Wellcome Trust (107469/Z/15/Z), British Heart Foundation (RE/18/4/34215, FS/IPBSRF/22/27059), and the National Institute for Health and Care Research (NIHR) Imperial College Biomedical Research Centre. Part of this research was made possible through access to the data and findings generated by the 100 000 Genomes Project. The 100 000 Genomes Project is managed by Genomics England Limited (a wholly owned company of the Department of Health and Social Care). The 100 000 Genomes Project is funded by the NIHR and National Heart Service (NHS) England. The Wellcome Trust, Cancer Research UK and the Medical Research Council have also funded research infrastructure. The 100 000 Genomes Project uses data provided by patients and collected by the NHS as part of their care and support. The UK Biobank recruited 500 000 participants aged 40 to 69 years across the UK between 2006 and 2010 (National Research Ethics Service, 11/NW/0382; 10.1371/journal.pmed.1001779). This study was conducted under terms of access approval number 47602. Written informed consent was provided.

### Disclosures

Dr Ochoa is an employee of Health in Code. Dr Ware has consulted for MyoKardia Inc, Foresite Labs, and Pfizer. The other authors reports no conflicts.
